# A Comparative Evaluation of the Primary and Secondary Stability of Dental Implants with Progressive and Conventional Thread Designs: A Prospective Non-Interventional Study of 100 Implants in 62 Patients

**DOI:** 10.3390/jcm14093040

**Published:** 2025-04-28

**Authors:** Daniel Seidel, Jörg Neugebauer, Günter Dhom, Octavio Weinhold, Kai-Peter Zimmermann, Robert Sader, Paul Weigl, Peter Gehrke

**Affiliations:** 1 Private Practice and Independent Researcher, 09117 Chemnitz, Germany; 2 Department for Oral and Craniomaxillofacial and Plastic Surgery, University Hospital Cologne, University of Cologne Faculty of Medicine, 50931 Cologne, Germany; 3 Transfer Institute Management of Dental & Oral Medicine, Steinbeis University, 39104 Magdeburg, Germany; 4 Private Practice for Oral Surgery and Implant Dentistry, 67059 Ludwigshafen, Germany; 5 Department for Oral, Cranio-Maxillofacial and Facial Plastic Surgery, Medical Center, University Hospital, Goethe University Frankfurt, 60596 Frankfurt, Germany; 6 Department of Postgraduate Education, Center for Dentistry and Oral Medicine (Carolinum), University Hospital, Goethe University Frankfurt, 60596 Frankfurt, Germany

**Keywords:** implant macro-design, thread design, primary stability, secondary stability, implant stability quotient (ISQ), resonance frequency analysis (RFA)

## Abstract

**Objectives**: We wished to compare the primary and secondary stability of dental implants with a progressive design (PL) versus a conventional thread design (SL) across various clinical settings. **Methods**: A total of 100 implants (50 PL and 50 SL) were placed in 62 patients. The stability of the implants was assessed using a resonance frequency analysis (RFA) at the time of placement (T1) and 20 weeks postoperatively before prosthetic loading (T2). Bone density was measured in Hounsfield units (HU) using cone-beam computed tomography (CBCT). The ISQ values were recorded for each group and anatomical region, including both inter- and intragroup comparisons over time. **Results**: Both implant designs showed a significant increase in stability during the healing period. At T1, the ISQ values were comparable between groups (SL: 71.3 ± 8.6; PL: 71.1 ± 8.7). At T2, the ISQ values increased significantly in both groups (SL and PL: *p* < 0.01), with no statistically significant difference in the degree of the gain in stability. The ISQ values were generally lower in the maxilla compared to those in the mandible. In the posterior mandible, the SL implants demonstrated a greater increase in stability compared to that with the PL implants. A strong positive correlation between the HU and ISQ values was observed for both groups (SL: r = 0.95; PL: r = 1.00), without reaching statistical significance. **Conclusions**: While the progressive thread design aims to enhance the primary stability, it did not outperform the conventional design in this study. Both implant types proved effective in achieving stable and predictable clinical outcomes.

## 1. Introduction

Long-term clinical studies report consistently high success rates for dental implants in both partially and fully edentulous jaws [1,2]. However, implant failure remains a relevant concern, particularly in regions with poor bone quality, such as the maxillary region [3]. A systematic review with a meta-analysis revealed that the early implant failure rates are higher in the maxilla compared to those in the mandible [4]. The initial implant stability, defined as the mechanical engagement of the implant with the surrounding bone at the time of placement, is a key determinant of successful osseointegration and clinical outcomes. This primary stability is influenced by several factors, including bone quality and volume, the implant’s geometry and morphology, the surgical technique, and insertion torque [5,6]. As new bone forms around the implant, biological fixation occurs, leading to secondary stability. Insufficient primary stability can result in micromovements which, if they exceed a critical threshold, may interfere with bone remodeling and compromise osseointegration [7]. Implant stability serves as a predictive parameter for osseointegration and long-term treatment success. Measuring the implant stability at different time points provides insight into the healing process and can guide decisions regarding the appropriate loading protocols. Resonance frequency analysis (RFA) is a widely used, non-invasive method that quantifies stability through the implant stability quotient (ISQ) [8]. The ISQ reflects the stiffness of the bone–implant interface and is reported on a scale from 0 to 100, with higher values indicating greater stability. RFA is a diagnostic method that makes use of a piezoelectric transducer to cause vibrations in an implant by emitting a sinusoidal signal at a particular frequency. The instrument measures the implant’s resistance to this vibration, converting it into an implant stability quotient (ISQ) on a scale from 0 to 100, where 100 represents the maximum implant stability [9].

The design of a dental implant includes both micro- and macro-geometric features, with each contributing differently to the osseointegration process [10]. Micro-design refers to the surface topography and texture, which influence cellular behavior and the bone response [11,12]. These features play a significant role in enhancing secondary stability. In contrast, macro-design elements, such as the implant’s shape, diameter, and length and the thread geometry, primarily affect the initial mechanical stability by promoting the optimal engagement with the host’s bone [13].

An implant’s shape is designed to distribute occlusal forces efficiently and reduce shear stress. Among the common designs, tapered and cylindrical implants have shown a superior performance in low-density bone by enhancing bone condensation and the stress distribution compared to these properties with cylindrical implants [14]. However, clinical comparisons have revealed a similar primary stability for tapered and cylindrical implants when they are placed in the posterior mandible, suggesting that the anatomical location may modulate the effect of the implant’s shape [15]. The thread design is a key macro-geometric feature that influences the mechanical retention and load distribution [16]. Conventional thread designs aim to distribute the stress along the implant body. Progressive thread designs, in contrast, are intended to enhance the primary stability by optimizing the thread depth, pitch, lead, and helix angle [10,17]. A smaller thread pitch increases the surface area and bone contact, potentially improving the primary stability, while a larger pitch reduces the insertion torque but may compromise initial engagement.

Thread configurations may be single-, double-, or triple-thread, with distinct biomechanical behaviors [16]. Single-thread designs offer a gradual insertion path and are often favored in soft bone [18]. Double-thread implants allow for faster insertion and may provide a more uniform stress distribution in denser bone. Triple-thread designs further reduce the insertion times but can compromise control over the placement and have been associated with an increased risk of peri-implantitis if not handled with care [19].

To address the challenge of achieving reliable primary stability in low-density bone and to meet the demand for early or immediate loading, implant manufacturers have introduced various modifications to the thread and body geometries. Despite their theoretical advantages, there is limited clinical evidence directly comparing conventional and progressive thread designs in terms of implant stability.

The present study therefore sought to evaluate the impact of two implant designs on primary and secondary stability. The two implant designs were as follows: one with a parallel, slightly tapered shape and a single-thread configuration and another with a progressive thread and an apically tapered shape. The stability of the implant was assessed using an RFA at two time points: immediately after the implant placement (T1) and prior to prosthetic loading (T2). The hypothesis tested was that the two implant designs would exhibit distinct mechanical and clinical performance profiles during the healing phase.

## 2. Materials and Methods

### 2.1. The Study Design and Population

Between October 2020 and April 2022, patients seeking implant treatment at a private clinic (D.S.) were screened and recruited for this prospective, non-interventional study based on defined inclusion and exclusion criteria. All participants received professional oral hygiene support and were informed in detail about this study’s objectives and procedures. Written informed consent was obtained from each participant. Eligible patients were aged 21 years or older, presented with one or more missing teeth in the mandible or the maxilla, and required either single or multiple adjacent implant restorations. The implant sites were required to have an adequate alveolar bone volume without the need for major augmentation procedures. To standardize the surgical complexity, patients requiring large bone grafts or sinus lifts were excluded. Further exclusion criteria included poor oral hygiene, untreated caries and periodontitis, systemic conditions that could compromise healing, and a history of bisphosphonate or corticosteroid medication use. Additional exclusion criteria included head and neck radiation therapy, pregnancy or breastfeeding, heavy smoking, bruxism, and substance abuse. The preoperative evaluation included a thorough clinical examination and cone-beam computed tomography (CBCT) of the planned implant region. All CBCT scans were acquired using standardized imaging parameters. Bone density was assessed in Hounsfield units (HU) using OnDemand 3D software (Version 1.0.10.5385, Cybermed Inc., Seoul, Republic of Korea), according to the Misch bone density classification [20]. A recent systematic review supports the use of CBCT gray values as a reliable estimate of bone density prior to implant procedures [21].

The study protocol was conducted in accordance with the Declaration of Helsinki and approved by the Ethics Committee of the State of Rhineland-Palatinate, Germany (file number 2023-17012).

### 2.2. The Clinical Protocol

**Figure 1:**
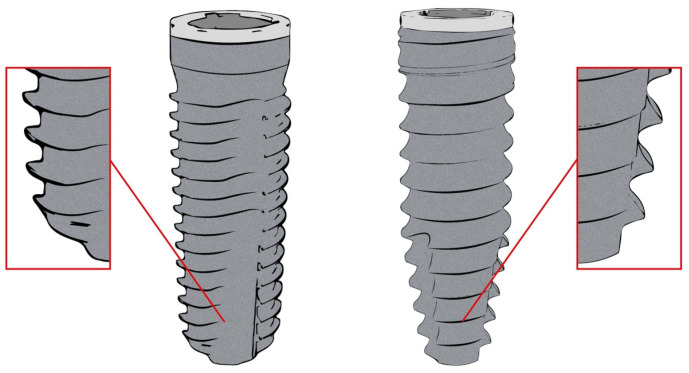
A depiction of the two implant designs assessed. Left: The SL implant with a slight taper and a single thread with a 0.7 mm uniform pitch. Right: The PL implant with a parallel-walled, apically tapered shape, featuring a 1.0 mm uniform thread pitch and crestal micro-threads at the top.

A total of 100 implants were placed and randomly assigned into two groups using a computer-generated sequence (Excel RAND function). This method ensured concealed allocation, although operator blinding was not applied. One group (*n* = 50) received implants with a parallel, slightly tapered shape and a single thread design with a uniform pitch of 0.7 mm (Screw-Line, Camlog, Wimsheim, Germany). The second group (*n* = 50) received implants with a progressive thread design (Progressive-Line, Camlog, Wimsheim, Germany), characterized by a combination of parallel walls and a tapered apical section with a uniform thread pitch of 1.0 mm and a crestal micro-thread at the top of the implant (Figure 1). Both implant types were fabricated from grade 4 titanium, featured identical prosthetic internal tube-in-tube connections, and shared a grit-blasted, acid-etched surface (Promote Plus, Camlog, Wimsheim, Germany). The collar of each implant included a 0.4 mm machined margin.

All surgical procedures were performed at healed sites by a single experienced clinician (D.S.) using a submerged technique, following the manufacturer’s recommendations. In cases requiring minor augmentation, guided bone regeneration (GBR) was performed using a xenogeneic grafting material (Bio-Oss Collagen, Geistlich Pharma AG, Wolhusen, Switzerland). The implant dimensions were selected based on individuals’ bone volume and quality. Implant stability was assessed using an Osstell Beacon (W&H Dentalwerk Bürmoos GmbH, Bürmoos, Austria). The corresponding transducer (SmartPeg, W&H Dentalwerk Bürmoos GmbH, Bürmoos, Austria) was hand-tightened into the implant’s internal connection using a dedicated driver. Measurements were obtained from the buccal/lingual and mesial/distal directions, and the mean ISQ value was recorded. Primary stability (PS) was measured immediately after implant placement (T1), and secondary stability (SS) was assessed approximately 20 weeks later, prior to prosthetic loading (T2). All of the ISQ measurements were performed by the same investigator (D.S.). To reduce measurement bias, the baseline clinical and patient-specific data were blinded during the stability assessments. At T1, the clinical evaluation included an assessment of pain, palpation, removal of the healing abutment, and the ISQ measurements. Implants showing clinical mobility, signs of infection, or unusual postoperative pain were excluded.

### 2.3. The Statistical Analysis

The data analysis was performed using SPSS software (version 22.0; SPSS Inc., Chicago, IL, USA). The significance level was set at *p* < 0.05. Descriptive statistics were used to summarize demographic and clinical variables. The Shapiro–Wilk test was applied to assess the normality of the data distribution. Depending on the data’s characteristics, the results are presented as means with standard deviations or counts. Intergroup comparisons of the mean ISQ values at T1 and T2 were conducted using independent *t*-tests. A Pearson’s correlation analysis was used to assess the relationship between bone density (HU) and implant stability (ISQ values). A post hoc power analysis was performed using G*Power 3.1. For the overall sample (*n* = 100), this study achieved 93.4% power to detect a mean ISQ difference of 3.5 at α = 0.05.

## 3. Results

### 3.1. Patients’ Demographics, Implant Locations, and Healing Times

**Figure 2:**
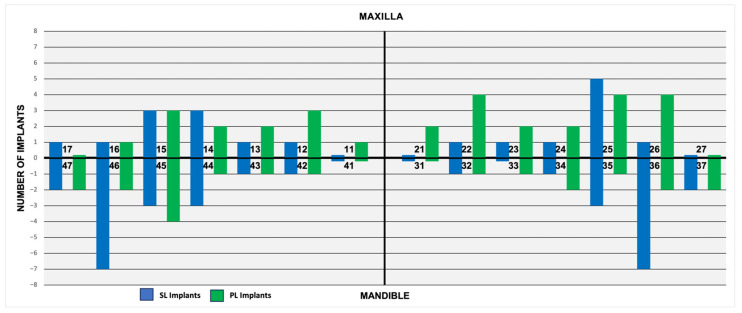
Distribution and locations of Screw-Line (SL, blue) and Progressive-Line implants (PL, green).

A total of 62 patients (26 women and 36 men; a mean age of 68.8 years ± 12.1 years, range: 21 to 88 years) received 100 dental implants which were subsequently restored with single implant-supported crowns (SICs) between October 2020 and April 2022. Of these, 50 implants had a conventional thread design (Screw-Line, SL), and the remaining 50 implants had a progressive thread design (Progressive-Line, PL). SL implants were placed in 32 patients (16 women, 16 men), while PL implants were placed in 30 patients (10 women, 20 men). The implants were distributed across the maxilla and the mandible based on individual treatment needs (Figure 2). In the SL group, 19 implants were placed in the maxilla and 31 were placed in the mandible, while 30 PL implants were inserted in the maxilla and 20 were placed in the mandible. Despite this variation, the implant distribution was comparable between groups.

The implant diameters ranged from 3.3 to 4.3 mm, and their lengths varied from 9 to 11 mm (Table 1). The variation in the implants’ diameters and lengths was within clinically acceptable limits, ensuring comparability across the groups and reflecting the routine variability in patients’ anatomy. The majority of the implants were placed in D3 bone (HU 351–850) according to the Misch classification (SL: *n* = 31; PL: *n* = 34), followed by D4 bone (SL: *n* = 17; PL: *n* = 11). The bone density could not be reliably assessed in six cases due to CBCT motion artifacts (Table 2).

Table 3 and Table 4 present the mean ISQ values by implant type (SL vs. PL), bone density (in Hounsfield units), diameter, and region of insertion, along with the total number of implants per group. Both the SL and PL implants exhibited similar primary stability (ISQ values) across different bone densities (D3, D4, and D5). The majority of the implants were placed in D3 (HU 351–850) and D4 (HU 150–350) bone. No implants were placed in D1 or D2 bone, and only one Screw-Line (SL) implant was inserted into D5 bone (HU < 150). The mean ISQ values increased with bone density, ranging from 75.0 in D5 to 80.5 in D3 for the SL implants and from 78.8 in D4 to 79.0 in D3 for the Progressive-Line (PL) implants.

The Pearson’s correlation analysis revealed a strong positive correlation between the bone density and ISQ values (SL: r = 0.95, *p* = 0.204; PL: r = 1.00, *p* = 1.00). However, statistical significance was not attained due to the limited sample size. The mean healing time, defined as the period between implant insertion (T1) and the final ISQ assessment prior to prosthetic loading (T2), was 21.8 ± 4.3 weeks. The SL group had a mean healing time of 21.1 ± 4.3 weeks, while the PL group averaged 22.5 weeks ± 3.6 weeks. No postoperative complications were observed, and the healing was uneventful in all cases.

### 3.2. The Analysis of the Implants’ Stability

**Figure 3:**
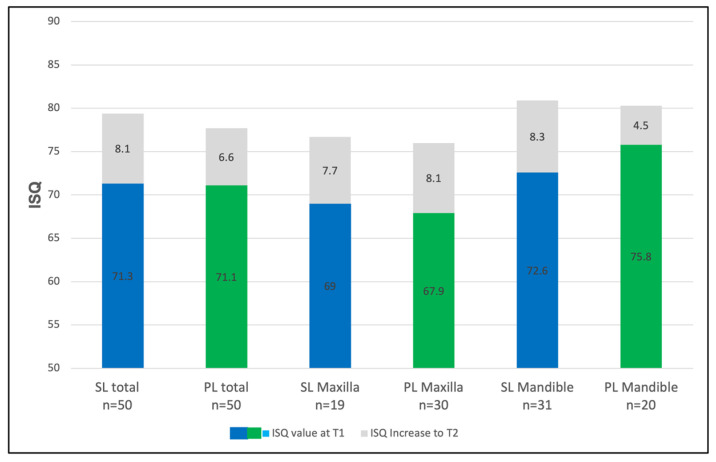
The mean implant stability quotient (ISQ) values in the maxilla and mandible at implant insertion (T1) for Screw-Line (SL, blue) and Progressive-Line (PL, green) implants. The ISQ increase observed until prosthetic loading (T2) is represented in gray.

At the baseline (T1), the overall mean implant stability across all implants was 71.2 ± 8.6 ISQ. The SL implants demonstrated a mean ISQ of 71.3 ± 8.6, while the PL implants showed an ISQ of 71.1 ± 8.7 (Figure 3). Prior to prosthetic loading (T2), the mean ISQ increased to 78.5 ± 4.8 overall, with values of 79.3 ± 4.0 in the SL group and 77.7 ± 5.4 in the PL group (Figure 3). The differences between groups were not statistically significant at either T1 (*p* = 0.90) or T2 (*p* = 0.09). However, the within-group comparisons revealed significant increases in the implants’ stability over time (SL: *p* < 0.01; PL: *p* < 0.01).

**Figure 4:**
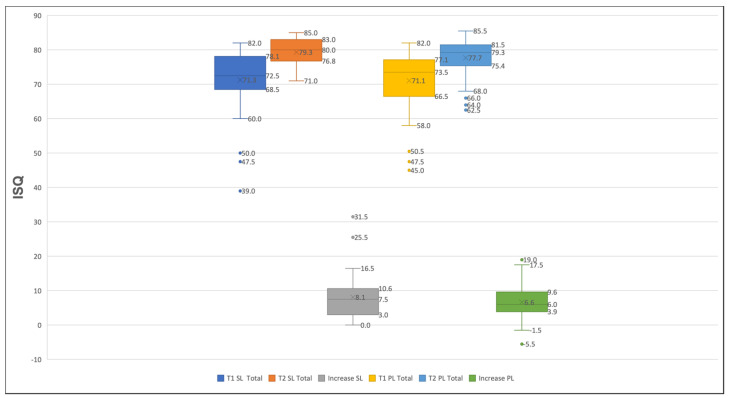
A Boxplot is showing total implant stability quotient (ISQ) values at implant insertion (T1) and at prosthetic loading (T2) for Screw-Line (SL) and Progressive-Line (PL) implants. Separate ISQ increases over time are displayed in gray for SL implants and in green for PL implants.

The mean ISQ gain was 8.1 points for the SL implants and 6.6 points for the PL implants (Figure 4). No significant difference was found between the groups regarding the overall gain in stability (*p* = 0.23).

### 3.3. The Regional Implant Stability

**Figure 5:**
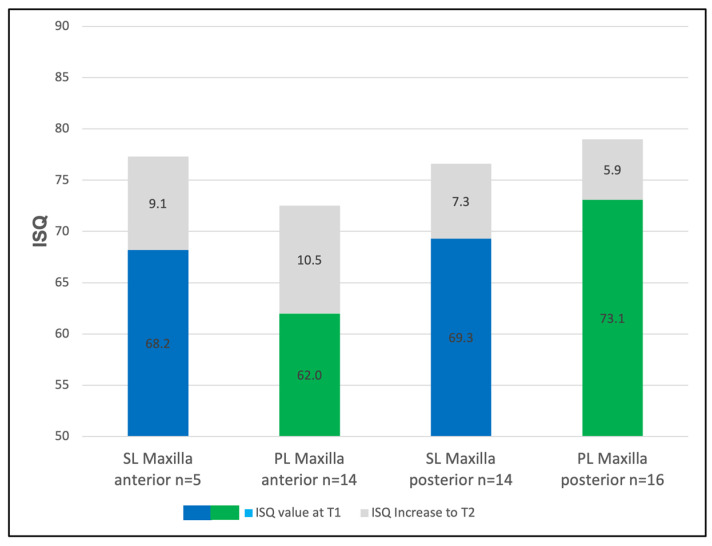
The mean implant stability quotient (ISQ) values in the anterior and posterior maxilla at implant insertion (T1) for Screw-Line (SL, blue) and Progressive-Line (PL, green) implants. The ISQ increase observed until prosthetic loading (T2) is represented in gray. The x-axis represents the location and time point (anterior–T1/T2, posterior–T1/T2), while the y-axis shows the ISQ values.

In the maxilla, the ISQ values increased from 69.0 to 76.7 for the SL implants (Δ = +7.7) and from 67.9 to 76.0 for the PL implants (Δ = +8.1). In the mandible, the ISQs of the SL implants rose from 72.6 to 80.9 (Δ = +8.3), while those of the PL implants increased from 75.8 to 80.3 (Δ = +4.5) (Figure 3). The individual analysis of the implant stability for the maxilla and the mandible yielded the following values: In the anterior maxilla (canine-to-canine), the SL implants showed a primary stability of 68.2 (±3.3) ISQ units, which increased to 77.3 (±3.3) ISQ units at T2 (Figure 5). The PL implants exhibited an initial stability of 62.0 (±9.5) ISQ units, which rose to 72.5 (±5.8) ISQ units at T2. In the posterior maxilla (the premolar and molar regions), the SL implants demonstrated a primary stability of 69.3 (±7.8) ISQ units at T1, which increased to 76.5 (±3.9) ISQ units at T2, indicating an increase of 7.2 units. The PL implants in this area exhibited an initial value of 73.1 (±6.2) ISQ units, which increased to 79.0 (±2.9) ISQ units at T2, representing an average increase of 5.9 units (Figure 5). The implementation of a segment-specific statistical analysis was hindered by disparities in the case numbers between the maxillary and mandibular segments (anterior/posterior) and the limited sample sizes in certain cases. The comparison between the SL and PL groups showed no statistical significance at either T1 or T2 (T1: SL vs. PL: *p* = 0.9; T2: SL vs. PL: *p* = 0.09).

**Figure 6:**
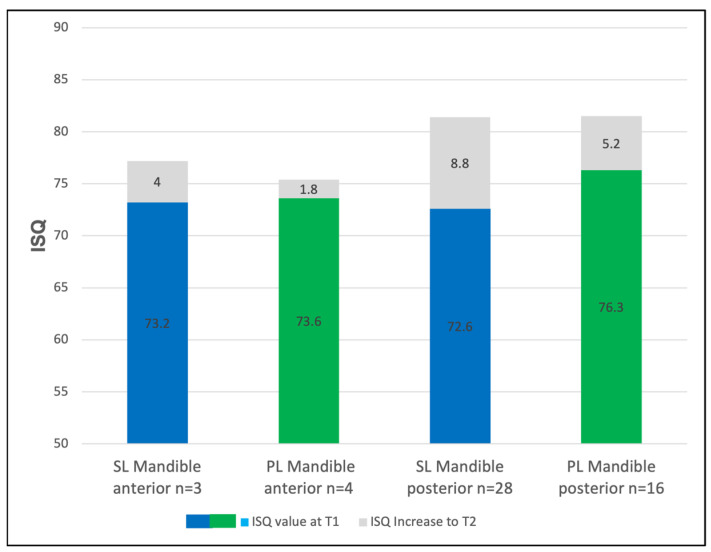
The mean implant stability (ISQ) values in the anterior and posterior mandible at implant insertion (T1) for Screw-Line (SL, blue) and Progressive-Line (PL, green) implants. The ISQ increase observed until prosthetic loading (T2) is represented in gray.

In the anterior mandible, the ISQs of the SL implants increased from 73.2 ± 5.6 to 77.2 ± 1.4, and those of the PL implants increased from 73.6 ± 5.4 to 75.4 ± 5.7 ISQ. In the posterior mandible, the SL implants showed a gain from ISQs of 72.6 ± 9.8 to 81.3 ± 3.2 (Δ = +8.7), while those of the PL implants rose from 76.3 ± 3.9 to 81.5 ± 2.8 (Δ = +5.2) (Figure 6).

## 4. Discussion

This prospective, non-interventional study assessed the influence of two different implant configurations on their primary and secondary stability. Stability was measured using a resonance frequency analysis (RFA) at the time of implant placement (T1) and before prosthetic loading (T2). Overall, both implant designs showed significant improvements in the ISQ values over the healing period. Although the PL design aims to enhance the initial mechanical retention, our findings demonstrated no significant difference in the primary stability between the two implant types at the baseline. Similarly, the mean gain in the ISQs from T1 to T2 was comparable between the groups, suggesting that the proposed advantages of the progressive thread geometry were not clinically evident under the conditions of this study. Therefore, the hypothesis that the two implant designs would exhibit distinct mechanical and clinical performance profiles during the healing phase must be rejected. The mean ISQ values were lower in the maxilla than those in the mandible, reflecting the generally lower bone density in the maxillary region. This pattern aligns with previous reports highlighting the influence of bone quality on implant stability [22,23]. Despite the anatomical variability, both the SL and PL implants demonstrated reliable osseointegration, as evidenced by the significant increase in the ISQs within each group.

A more detailed analysis by anatomical region revealed minimal and clinically negligible differences between the SL and PL implants in the anterior maxilla, posterior maxilla, and anterior mandible. These findings challenge the assumption that progressive thread designs provide a clear advantage in areas with a lower bone density, such as the posterior maxilla [24]. Interestingly, in the posterior mandible, a slightly higher increase in the ISQs was demonstrated for the SL implants compared to that for the PL implants. While both designs reached similarly high ISQ values at T2, the greater gain in the ISQs in the SL group may suggest that in dense bone regions, conventional thread geometries perform equally or even more favorably than more complex thread designs. These results are in line with those reported by Waechter et al. [15], who found similar biological responses for tapered and cylindrical implants in posterior mandibular sites, emphasizing the importance of the bone site characteristics over that of the implant geometry alone.

The implant diameters and lengths showed routine clinical variations and were not standardized due to ethical and anatomical considerations. Previous studies have reported conflicting results regarding the influence of the implant dimensions on primary stability. While some suggest that an increased diameter improves the mechanical engagement [25], others found no significant effect [26,27,28]. In the present study, both designs showed comparable stability trends across different diameters and lengths, supporting the notion that the surgical technique and bone quality may outweigh dimensional factors in determining the initial stability.

Recent evidence highlights the impact of the surgical site preparation techniques on the primary implant stability, particularly in low-density bone [29]. Osseodensification has been shown to increase the bone-to-implant contact and improve the mechanical retention by compacting rather than removing bone during osteotomy preparation [30,31,32]. Similarly, manual bone compaction may enhance the implant stability by preserving the trabecular architecture and promoting greater cortical engagement [33]. These techniques may be especially beneficial when combined with thread designs optimized for low-density bone, and future studies should explore their integration into the clinical protocols.

While this study focused on primary and secondary stability, it is important to recognize the influence of marginal bone loss (MBL) on long-term implant success. MBL has been associated with biomechanical stress, peri-implant inflammation, and implant design features [34,35]. Primary stability is a prerequisite for osseointegration, but sustained peri-implant bone levels are essential for functional and esthetic outcomes. Piezoelectric surgery, which allows for precise and minimally traumatic bone preparation, has not demonstrated superiority to conventional drilling in terms of the implant stability during the healing period [36]. Likewise, manual bone compaction techniques have demonstrated beneficial effects on primary stability by increasing the bone–implant contact and reducing implant micromotion [13,37]. Collectively, these findings underscore the multifactorial nature of primary stability and support a combined strategy that aligns the implant macro-geometry with site-specific surgical techniques to optimize the clinical outcomes.

Despite variations in the thread geometry, the differences in the primary stability between the examined implant systems appear minimal when appropriate implant site preparation techniques are employed [30]. Studies by Ho et al. and Kim et al. similarly concluded that while the thread design may influence the insertion characteristics and the initial torque, it does not necessarily result in superior RFA-derived stability values [38,39]. Yamaguchi et al. found that although double-threaded implants initially exhibited higher stability in terms of the insertion and removal torque, single-threaded implants with a reduced pitch and lead demonstrated comparable or even superior stability as measured using the ISQ [18]. Moreover, recent findings highlight the need for implant system-specific calibration of the ISQ values to allow for valid comparisons [40]. In the current study, all measurements were performed using consistent SmartPegs and by the same examiner, thereby minimizing potential variability [41].

Careful consideration of the limitations of this study and their potential impact on its results should be made. Resonance frequency analysis (RFA) is widely accepted as a non-invasive, objective, and reliable method for assessing implant stability [42,43]. However, the approximately one-week discrepancy in the mean healing times between the SL and PL implant groups may have influenced the secondary stability measurements. This variation is considered relatively minor and unlikely to have affected the outcomes given that osseointegration is typically well established after 12 to 16 weeks [44]. A notable limitation of this study is the absence of insertion torque data, which could have provided additional insights into the mechanical engagement between the implants and bone. Although ISQ values offer useful information about implant stability, insertion torque reflects a different biomechanical aspect of implants’ primary stability. Previous studies have shown that the insertion torque and ISQ values do not always correlate, suggesting that both parameters should be assessed for a comprehensive evaluation of implant stability [27,45]. Future studies should integrate both RFA and torque-based measurements for a more nuanced analysis.

The post hoc analysis confirmed that the overall sample size was sufficient to detect clinically relevant differences in the ISQ values. However, the regional subgroup analyses (e.g., posterior maxilla) showed a substantially lower statistical power (47.0%), indicating a limited ability to detect smaller differences in the implant stability in the anatomical subgroups. The use of computer-generated randomization to assign the implants into groups may be another limitation, as the lack of operator blinding may have affected the internal validity of the results.

Although a positive correlation between the bone density (HU) and ISQ values was observed, statistical significance was not reached, likely due to the limited sample size and the underrepresentation of high-density bone (D1 and D2). As most of the implants were placed into D3 and D4 bone, these findings may not be generalizable to patients with very dense bone. Nevertheless, future studies should aim to include larger and more balanced cohorts to validate these findings across a broader range of bone qualities and clinical conditions.

## 5. Conclusions

Within the limitations of this study, both the conventional (SL) and the progressive (PL) implant designs exhibited a comparable performance in terms of their primary and secondary stability across different bone densities and anatomical regions. While the progressive thread configuration was designed to enhance primary stability, its clinical benefits did not surpass those of the conventional design in this investigation. These findings underscore that implant stability is influenced by multiple factors, including the surgical protocol, bone quality, and clinician experience. The results support the clinical reliability and predictability of both implant designs in routine implant therapy. Future research involving larger samples, the inclusion of insertion torque data, and extended follow-up periods are needed to clarify the specific impact of the thread geometry on the implant stability and long-term treatment outcomes.

## Figures and Tables

**Table 1:** The dimensions of the inserted Screw-Line (SL) and Progressive-Line (PL) implants (diameter and length in millimeters).

Implant Dimensions	3.3 × 11	3.3 × 13	3.8 × 9	3.8 × 11	3.8 × 13	4.3 × 9	4.3 × 11	4.3 × 13	Total *n*=
*n* = SL ^1^	-	-	6	4	10	10	18	2	50
*n* = PL ^2^	2	2	1	15	11	6	12	1	50

^1^ Screw-Line; ^2^ Progressive-Line.

**Table 2:** Bone density assessment at implant site according to bone classifications D1-D5 for Screw-Line (SL) and Progressive-Line (PL) implants.

Bone Density	SL Implants ^1^	PL Implants ^2^
D1	0	0
D2	0	0
D3	31	34
D4	17	11
D5	1	0
Not evaluable	1	5
Total	50	50

^1^ Screw-Line; ^2^ Progressive-Line.

**Table 3:** Mean ISQ values by implant type (Screw-Line vs. Progressive-Line) and bone densities D3 to D5 in Hounsfield units (total implants).

Bone Density (Hounsfield Units)	SL Implants ^1^	PL Implants ^2^
D5 (HU < 150)	75.0 (*n* = 1)	(*n* = 0)
D4 (HU 150–350)	78.2 (*n* = 17)	78.8 (*n* = 11)
D3 (HU 351–850)	80.5 (*n* = 32)	79 (*n* = 34)

^1^ Screw-Line; ^2^ Progressive-Line.

**Table 4:** Mean ISQ values by implant type, diameter, and region of insertion (total implants). Screw-Line (SL) and Progressive-Line (PL) implants.

Implant Type and Diameter	Anterior Maxilla	Posterior Maxilla	Anterior Mandible	Posterior Mandible
SL ^1^ D3.8 mm	78.2 (*n* = 3)	74.8 (*n* = 6)	77.2 (*n* = 3)	79.8 (*n* = 8)
SL ^1^ D4.3 mm	76.0 (*n* = 2)	77.9 (*n* = 8)	(*n* = 0)	82.0 (*n* = 20)
PL ^2^ D3.3 mm	69.8 (*n* = 4)	(*n* = 0)	(*n* = 0)	(*n* = 0)
PL ^2^ D3.8 mm	73.7 (*n* = 10)	80.2 (*n* = 6)	75.4 (*n* = 4)	80.0 (*n* = 7)
PL ^2^ D4.3 mm	(*n* = 0)	78.3 (*n* = 10)	(*n* = 0)	82.7 (*n* = 9)

^1^ Screw-Line; ^2^ Progressive-Line.

## Data Availability

The data presented in this study can be made available on reasonable request to the corresponding author.

## Abbreviations

The following abbreviations are used in this manuscript:

PL	Implants with progressive thread design
SL	Implants with conventional thread design
RFA	Resonance frequency analysis
ISQ	Implant stability quotient
T1	Time point immediately after implant placement
T2	Time point at prosthetic loading
CBCT	Cone beam computed tomography
HU	Hounsfield units
GBR	Guided bone regeneration
PS	Primary implant stability
SS	Secondary implant stability
